# Educational innovation: the architecture of digital technologies as a catalyst for change in university teacher training

**DOI:** 10.1038/s41598-023-48378-w

**Published:** 2023-11-28

**Authors:** Erick Flores-Chacón, Alex Pacheco, Yvett Gonzales-Ortiz, Lenin Moreno-Vega, Fiorella del-Castillo-Palacios, Even Perez-Rojas

**Affiliations:** 1 Universidad Cesar Vallejo, Lima, Peru; 2 Universidad Nacional de Cañete, Lima, Peru; 3 Universidad Nacional Santiago Antúnez de Mayolo, Huaraz, Peru

**Keywords:** Engineering, Electrical and electronic engineering

## Abstract

The aim of this research was to determine the impact of the architecture of digital technologies in university teacher training as a catalyst for change and educational innovation. Methodologically, it is framed in a quantitative approach with a pre-experimental design and an explanatory level, with a sample of 269 teachers out of a total population of 450 at the university. The results show a significant impact of the architecture of digital technologies as a catalyst for change in university teacher training, with an increase of 22.88% from pre-test to post-test. The statistical significance of the results is supported by a *P*-value of 0.000, which means that it is less than the established significance level (α = 0.05), leading to the acceptance of hypothesis H1: The architecture of digital technologies as a catalyst for change in university teacher education has an impact. In conclusion, the architecture of digital technologies, consisting of the two academic pillar systems: the academic management system and the virtual learning system, contribute to the development of digital skills in key areas such as digital literacy, communication and collaboration, digital content creation and problem solving in virtual environments.

## Introduction

The COVID-19 pandemic has led to the declaration of a global health emergency, creating a contradictory scenario of opportunities. On the one hand, there was an acceleration of digital transformation worldwide, especially in Latin American countries. These countries faced the challenge and accelerated the maturity process according to their dimensions, resources and cultural size^1^^,^^2^. On the other hand, educational institutions also faced a similar scenario. This created the need to train teachers in the management of the Virtual Learning System—Asynchronous (VLS) with innovative tools such as Moodle, in order to improve their performance in the development of teaching–learning sessions of virtualised courses.

The current context motivates to engage in the new relationship between human beings and education, known as Education 4.0, which is a teaching approach aligned with the fourth industrial revolution, characterised by the integration of advanced digital technologies. According to Celis, Trejo and Gómez^3^, the value of Education 4.0 lies in the proper integration of digital technologies in the classroom, along with the constant reflection of teachers on the technological, pedagogical and organisational aspects of their work. This requires strategic action on the part of teachers in order to achieve quality education that is relevant to the needs of today’s world^4^^,^^5^.

The common framework for digital literacy in teaching^6^ identifies five areas of competence that aim to improve and strengthen teachers' specific knowledge in their teaching area. These areas are related to technology and its application in teaching to meet the learning needs of today's students. They are: Area 1: Information and Information Literacy; Area 2: Communication and Collaboration; Area 3: Digital Content Creation; Area 4: Security; and Area 5: Problem Solving. All make intensive and strategic use of digital technologies.

In Peru, digital technologies have been formally defined as everything related to information and communication technologies (ICTs), including the Internet, mobile technologies and devices, as well as data analytics used to improve the generation, collection, sharing, aggregation, combination, analysis, access, search and presentation of digital content. This definition also includes the development of digital government services and applications, as identified by the Chair of the Council of Ministers. Furthermore, the Economic Commission for Latin America and the Caribbean^7^ points out that, in the current context, the rapid technological integration and convergence processes of different technologies will condition the development of ICTs. According to this proposal, the following dimensions related to digital technologies can be structured: networks (cable technology and mobile networks), hardware devices (mobile multimedia devices), computing services and applications (cloud computing) and web technologies (web 2.0). It is important to stress that these dimensions must be strategically managed in order to facilitate the implementation, development and maturity of the information and knowledge society, so that every Peruvian citizen has full, inclusive and democratic access to the Fourth Industrial Revolution, which includes advanced digital technologies such as 5G technology, big data, data science, artificial intelligence and business intelligence.

In the Peruvian context, universities require an architecture of digital technologies for learning and development of digital competences that allows them to use cloud computing, the Learning Management System (LMS) based on Moodle and the Microsoft Teams tool^8^. Furthermore, there is a need to train teachers in the Virtual Learning System (VLS) with Moodle to improve their performance in online teaching. This architecture of digital technologies is very important for teachers to train, coach and develop their performance to improve their professional practice, mainly in the areas of digital technology, pedagogy^9^ and communication^10^ in the production of disciplinary content to advance the great challenge of Teacher 4.0, referring to educators who have adapted their skills and teaching methods to align with the demands of the fourth industrial revolution.

In particular, this situation is reflected in university teachers at the Universidad Nacional Santiago Antúnez de Mayolo who require a training programme in the management of the Virtual Learning System (VLS) with the Moodle tool, in order to improve their performance in the development of teaching–learning sessions of virtualised courses in response to the digital transformation accelerated by the COVID-19 pandemic. Therefore, the aim of this research is to determine the impact that the architecture of digital technologies have on university teacher training as a catalyst for educational change and innovation.

## Methods and materials

The present research is framed within a quantitative approach with a pre-experimental design and an explanatory level. The sample consisted of 269 teachers out of a total population of 450 at the Universidad Nacional Santiago Antúnez de Mayolo (UNASAM), to whom the Pretest and Posttest Survey on the didactic moments used for the training of the Virtual Learning System (SVA) was applied using the Google Forms tool, with the informed knowledge of the participants.

Various statistical analyses were then carried out: firstly, the reliability of the instrument was analysed using Pearson’s R Alternate Form Reliability Test model. Second, the Kendall’s W Concordance Coefficient model was used to analyse the validation of expert judgement. Thirdly, the Kolmogorov–Smirnov model was used for the normality analysis and finally the Wilcoxon model was used for the hypothesis validation analysis. All these analyses were carried out using SPSS 25 and MS-Excel statistical software tools.

In terms of the methods used to generate knowledge, the scientific method, the logical-deductive method and the indirect-deductive method were considered, which were combined and contributed to the research work and the generation of knowledge through documentary review and empiricism. Finally, the ethical aspects considered were respect for human beings and the search for the welfare of the research participants.

### Ethics approval and consent to participate

The present study was conducted in accordance with the ethical principles of the Declaration of Helsinki and was approved by the ethics committee of the company “Cocreando con Proposito SAC”. The workers understood the objectives and methods of the study and gave written informed consent.

## Results

### Academic model 4.0 proposed for Unasam

The Fourth Industrial Revolution represents a fundamental change in the way we live and work^11^. In this scenario, the strategies and implementation of Education 4.0 need to be considered. Based on this, the intersection of artificial intelligence, big data, constant connectivity and robotics has been proposed to completely transform teaching, assessment and learning management^12^.

**Figure 1:**
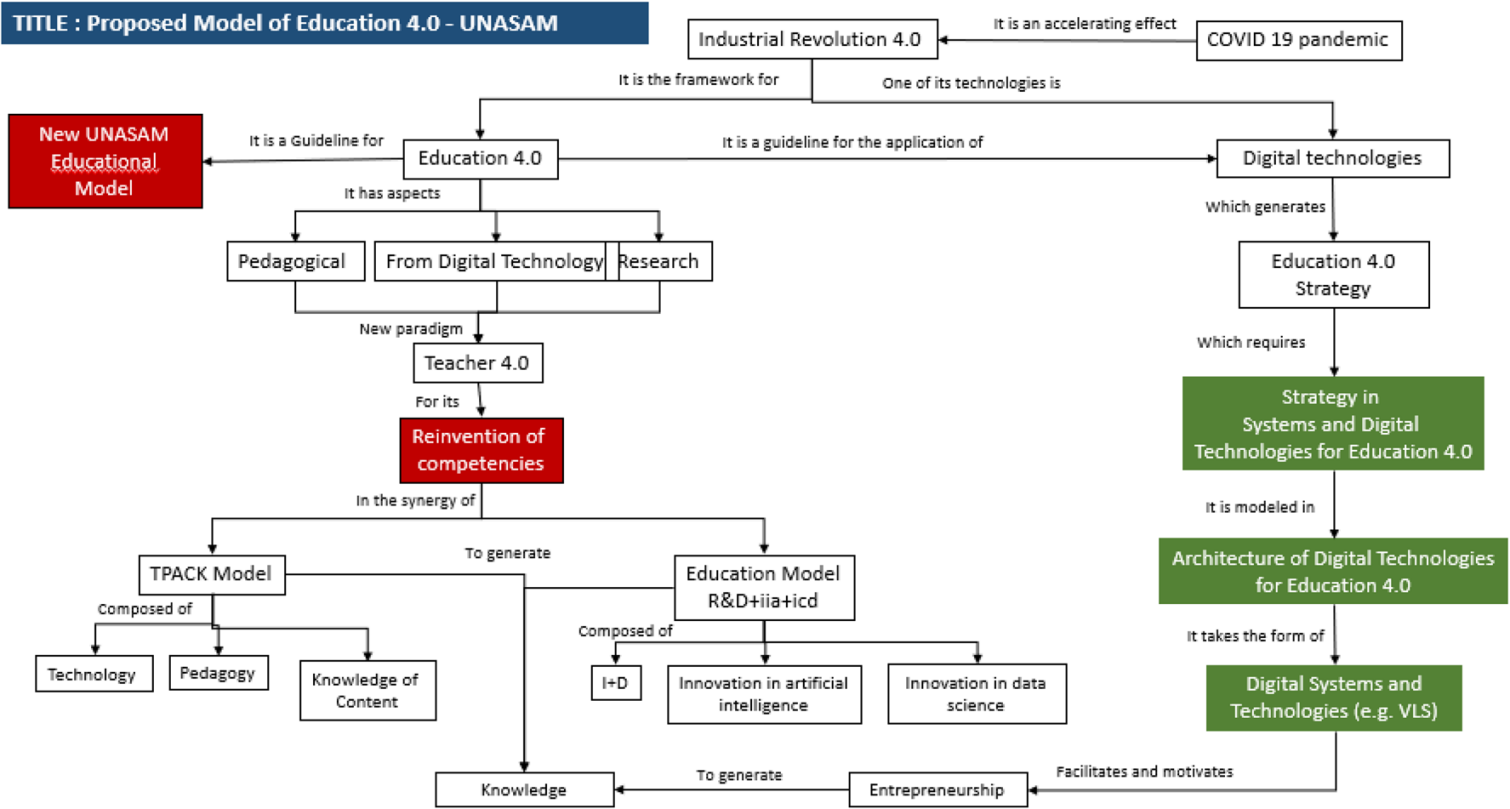
Academic Model 4.0—UNASAM. *Note* IR4.0 is the framework for Education 4.0, which is the guideline for the strategy of systems and digital technologies that contribute to the reinvention of competencies of Unasam's Teacher 4.0.

In this synergistic theoretical framework of topics such as the Industrial Revolution 4.0, Education 4.0 and digital technologies, the model of Education 4.0—Unasam is proposed as a model of reinvention of university teacher competencies facilitated by the strategy in systems and digital technologies for Education 4.0, as shown in Fig. 1.

### Architecture of digital technologies

**Figure 2:**
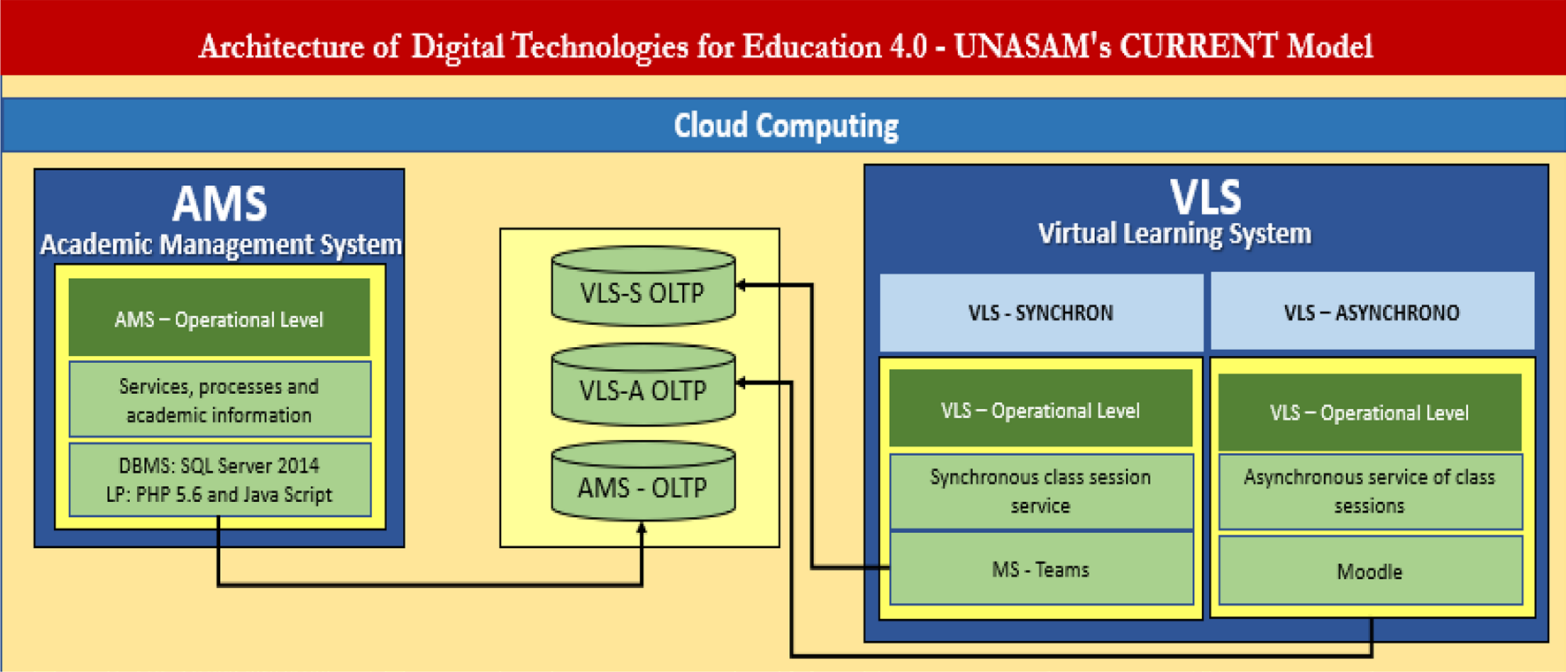
Architecture of digital technologies for Education 4.0—Unasam’s CURRENT model *Note* In each system the business level is highlighted, the services offered, as well as the software tools that produce and store the information in the OLTP databases. Empirical model of the academic management of the General Office of Studies.

Within the framework of the previous model, the architecture of digital technologies^8^ is designed for Education 4.0 with concrete solutions in digital systems and technologies such as: the Academic Management System (SGA) and the Virtual Learning System (SVA) implemented in the Moodle tool, detailed in Fig. 2.

**Figure 3:**
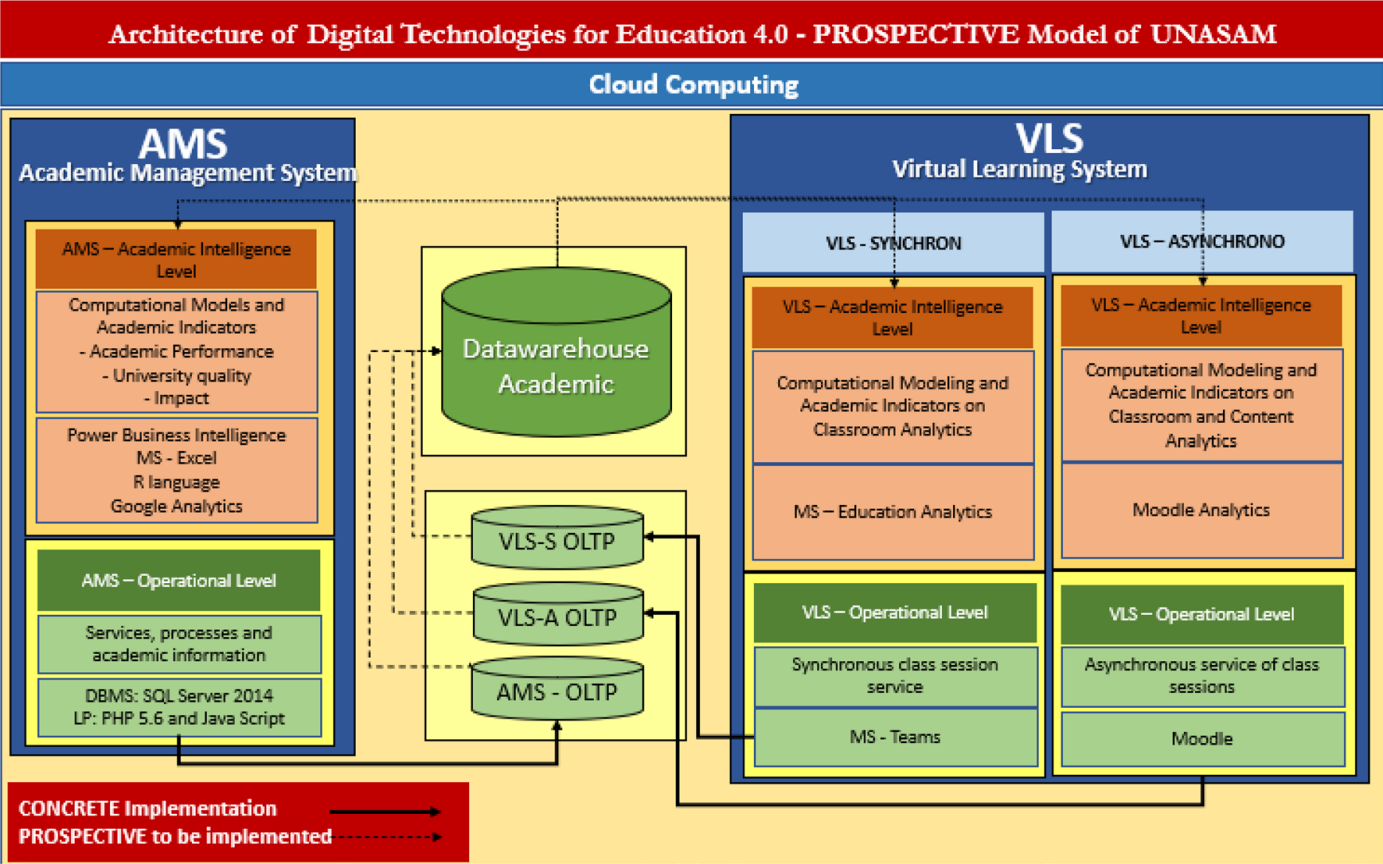
Architecture of digital technologies for Education 4.0—prospective model of the Unasam.* Note* Second floor to consolidate the Information and Knowledge Society and second floor, at the beginning of the Industrial Revolution 4.0 in Unasam.

In a prospective scenario of the strategy in systems and digital technologies for Education 4.0, the prospective architecture of digital technologies for Education 4.0 of Unasam was modelled^8^. This architecture is at the second level of the architecture shown in Fig. 2, with the aim of facilitating and motivating the generation of knowledge in the process of reinventing the competences of the Teacher 4.0. University teachers^13^ will be able to equip themselves with the necessary knowledge to improve the teaching–learning process through the synergistic use of digital technologies, pedagogy and disciplinary knowledge, achieving integration and assertive communication with students. Furthermore, in this process of reinvention, it is necessary to encourage research, development and innovation in the field of big data with artificial intelligence and data science, whether in disciplinary, pedagogical or other areas supported by the curriculum. Figure 3 illustrates this process.

### Teacher training in the virtual learning system (VLS)

The profile of today's teacher requires creative skills in planning, organising, designing, directing, guiding, in other words, managing all aspects of a learning experience in which disciplinary knowledge, digital technologies and the indispensable pedagogical conduit come together synergistically. The teacher's role is to train autonomous learners who are able to learn to learn, discriminate facts, identify and develop unconventional solutions, form opinions and defend them^14^.

Given the need to integrate digital technologies in the classroom, it is necessary to emphasise in the teacher training process the development of a set of competences in which the following technical and technological dimensions converge, leading to the appropriation of ICT and Web 2.0, to be able to defend oneself in a technological environment and to use it for life, taking advantage of its pedagogical potential: Disciplinary, refers to the importance of teachers reflecting on their own disciplinary training and their use of ICT. Pedagogical, it implies adopting a creative and innovative pedagogical perspective, using ICT to solve everyday problems and to carry out learning assessment processes. Inquiry, research processes are considered as curricular and pedagogical components of ICT in educational institutions. Attitudinal, related to motivational and affective processes that influence planning, attitudes and roles adopted in the use of technologies in the classroom. Teacher training and digital literacy. Communicative, which addresses the importance of establishing effective and multidirectional communication between students and teachers. Evaluative, which takes into account the need to combine different forms of evaluation^15^.

Unasam has had this architecture of digital technologies at the level of the second floor since 2017, an architecture that has helped to achieve the institutional licence by meeting the basic quality conditions required by SUNEDU. From the aforementioned date until the declaration of the pandemic by COVID-19, an average of 17% of teachers (of the approximately 450 teachers per academic semester) used the Virtual Learning System (SVA) for their teaching sessions.

The arrival of the pandemic accelerated the development of teachers’ digital skills, as they had to plan their courses, run their classes and assess students remotely and virtually, so the training model implemented developed digital and pedagogical skills through the Virtual Learning System (VLS) for asynchronous learning.

One of the strategies applied was the management and implementation of the Digital Training and Support Workshop for the Virtual Learning System (VLS) of the National University of Santiago Antúnez de Mayolo. This workshop, which is part of the theoretical-practical component of the VLS Moodle LMS training, covered a series of topics essential for the efficient use of the system. The content of the workshop included an overview of the VAS, providing participants with a comprehensive understanding of the platform. The general configuration of materials within the VAS was taught, as well as the organisation of teaching weeks, including the input of activities and resources such as teaching materials and video lectures. Specific settings for assignments and forums were also covered, as well as the integration of quizzes as part of the learning activities. Participants were also given the opportunity to practise and train with what had been developed to ensure understanding and the ability to apply the knowledge acquired. Guidance was given on setting up other activities and resources, such as files and URL links, and time was spent reviewing and recommending work submitted by learners, including marking. Finally, the workshop concluded with a general practice of the learning environment, allowing participants to consolidate their VAS skills and knowledge.

The topics were methodically and systematically structured according to the criteria of empathy and clear and simple communication for the users. Each session had a dynamic of systematised activities structured in four moments: a. development of the topic b. practice of the topic in the laboratory of the VAS training area c. feedback and accompaniment by the Digital Technologies team of the OGE at a defined time d. achievement and product of the session’s learning, evidenced in the VAS training area. All these aspects described above have been implemented and put into production in technological components of the University's digital technology architecture. The cloud computing service (hosting and processing in the cloud) of the service provider, the virtual learning system (VLS) through Moodle and the social network YouTube were used. All this consisted in carrying out the following steps: a. Methodological preparation of the learning session b. Elaboration and writing of the script for the video c. Recording of the video d. Editing of the video e. Quality control of the video and its content f. Production of the video on YouTube. Produce the video on YouTube. Producing the video on YouTube. Production of the video on YouTube g. Preparation of a manual that consolidates the workshop “Manual and Digital Workshop for Training and Support of the Virtual Learning System (SVA) of the National University Santiago Antúnez de Mayolo” in a systematic and orienting way. These procedures have been developed in the context of a strategic and prospective management of systems with digital technologies through managerial and professional competences (synergy between normativity, processes, pedagogy and systems with digital technologies innovated in the General Office of Studies—OGE).

### From the perspective of statistical results

#### Reliability test

The Alternate Form Reliability Test was used in this case. This measures reliability through the correlation between the two scores over a significant period of time between the two tests. Alternate, equivalent or parallel forms of the same test are two tests that are designed to be virtually the same, so that the scores of the subjects on both are comparable item by item. For reliability testing using Pearson’s R, the closer ‘r’ is to 1, the greater the relationship between the two instruments, indicating greater reliability. After applying Pearson’s R, under the Alternate Form model, before and after the application of the stimulus (pretest and posttest), r = 0.824789581 is obtained, which is considered excellent reliability.

#### Normality test

It should be confirmed that the random variable is normally distributed in both groups. The Kolmogorov–Smirnov K-S test is used for large samples (> 30 individuals), or the Shapiro–Wilk test when the sample size is < 30. The normality criterion is:

- *P*-value =  > α accept Ho = The data are from a normal distribution.
- *P*-value < α accept H1 = The data do not come from a normal distribution.

The* P*-Value was obtained = 0.003; which is < α (α = 0.05). *P*-value < α: Therefore; H1 = The data are not from a normal distribution is accepted.

#### Hypothesis validation test

This is the process of verifying the scientific knowledge of the theoretical-empirical model that needs to be corroborated from the data collected and which, by means of a statistical model, determines whether the hypothesis of the research is accepted or rejected. In the case of the present research, which is of a non-parametric, ordinal type with a longitudinal study, the test used was the Wilcoxon statistical model, applied to the research hypothesis.

##### Ho

The architecture of digital technologies does not improve teacher training in the VLS-Moodle of Unasam.

##### H1

The architecture of digital technologies improves teacher training in the VLS-Moodle of Unasam.

The statistical analysis that was performed at a significance level (alpha) α = 5% = 0.05. Taking into account the following rules:

If the obtained probability *P*-value ≤ α, reject Ho (H1 is accepted).

If the obtained probability *P*-value > α, do not reject Ho, (Ho is accepted).

*P*-value = 0.000 was obtained; which is < α (α = 0.05). *P*-value < α: Therefore; H1 = The architecture of digital technologies improves teacher training in the SVA-Moodle of Unasam is accepted.

#### Satisfaction with the training

**Figure 4:**
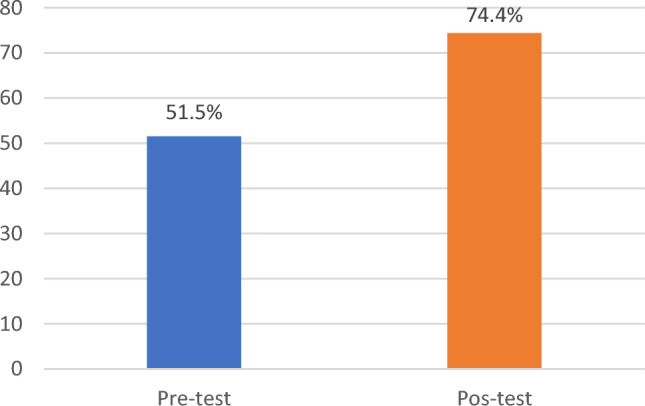
Teacher satisfaction with the asynchronous virtual learning system training.

We worked with a sample of 292 teachers who, with the information recorded in the pretest and posttest, collaborated with the instrument, Scale of Teacher Satisfaction with the Training in the Asynchronous Virtual Learning System of the National University Santiago Antúnez de Mayolo. The results show an improvement of 22.9% in the satisfaction with the training in the architecture of digital technologies, since in the pretest a result of 51.5% was obtained and in the posttest 74.4%. Figure 4 shows this.

## Discussion

The proposed model seeks to address the challenges that arose in the context of the COVID-19 pandemic, where universities and teachers had to adapt quickly to online education. Many teachers lacked the digital and pedagogical skills to meet this challenge and deliver quality online education. This is why the proposed holistic model aims to provide a clear structure for teacher training in the use of VAS and the Moodle platform, taking into account both technological and pedagogical aspects.

The model focuses on three main aspects: technical training, pedagogical training and management training. In terms of technical training, full training is provided in the use of the Moodle platform, from course creation to assessment and monitoring of student performance. Teachers are also trained in complementary digital tools that they can use to enrich the students' learning experience. This model builds on the findings of previous research by Chaparro-Diaz and Ballesteros-Ricaurte^16^, who characterised basic digital 2.0 competences in the use of the Moodle platform by a group of teachers in a technological educational institution. Both investigations support the need to integrate digital technologies in the education sector to promote the professional development of teachers, improve teaching processes and increase the quality of education.

The present research has shown that teachers improved their knowledge and skills in the use of ICT in their teaching–learning processes thanks to the strategic implementation of digital technology architecture. These results support the findings of Agustín et al.^17^, who point out that there is currently a low integration of ICT in teaching and learning processes, where assimilative and expository activities are prevalent. Furthermore, it is highlighted that ICT knowledge is an important competence in teacher professional development, which suggests that its integration is still limited in teaching and learning processes, and that traditional activities are still predominant.

Furthermore, the results of the proposed model highlight the importance of strategic systems management underpinned by the Digital Technology Architecture, in line with the recommendations of the Organisation for Economic Co-operation and Development (OECD)^18^. According to the OECD, to achieve successful results, digital technologies should be considered as an integral part of the curriculum approach, including teacher training and ICT support. This finding aligns with that of Tejedor et al.^19^, who note that teachers surveyed recognise the need to promote critical and reflective thinking in relation to the strategic management of ICT.

In order to achieve the objective of improving teacher training in the university's Virtual Learning System (VLS), a process of approach, induction and training was carried out with empathy, tolerance and accompaniment of the asynchronous VLS platform implemented in Moodle software, according to the university’s Digital Technology Architecture. The results obtained show a significant improvement in the satisfaction of teachers with the training in the architecture of digital technologies, increasing by 22.9% from the pre-test (51.5%) to the post-test (74.4%). This indicates that the objective of explaining how the digital technology architecture facilitates teacher training in the university’s VAS was met. The statistical significance of the results is supported by a *P*-Value of 0.000, which means that it is less than the established level of significance (α = 0.05), leading to the acceptance of hypothesis H1 that digital technology architecture enhances teacher training in the university's VAS.

## Conclusions

The conclusions of the work highlight the importance of the implementation of the Academic Model 4.0 of Unasam, which is based on the solution of the architecture of digital technologies and cloud computing technology for university education 4.0. This model is composed of two academic pillar systems: the Academic Management System (SGA) and the Virtual Learning System (SVA), which are fundamental to improve the quality of education and teaching processes in the digital era.

In addition, the importance of training university teachers in the use of these tools is highlighted as a necessary first step for their professional development in this new educational scenario. But it also emphasises the need for the reinvention of competences for the university teacher 4.0 in the future, as digital technologies are transforming the way in which learning and teaching takes place today. On the other hand, the research results favour the continuity of a second stage of the digital technological architecture in the guideline and framework for the development of academic intelligence based on Big Data, Data Science, Artificial Intelligence, Internet of Things and Business Intelligence. In this way, it seeks to further improve the quality of education and teaching processes through the implementation of innovative technologies and the continuous training of teachers in their use.

Based on the findings of this research, educators and schools could implement more effective practices to improve teacher training in the use of digital technologies. This could include adopting a robust and flexible technology architecture that allows teachers to explore and adapt to different online tools and resources asynchronously. Educational institutions could invest in e-learning platforms that offer intuitive interfaces and access to a wide range of interactive learning materials, which could significantly increase the satisfaction and effectiveness of teacher training. In addition, professional development programmes could include specific modules focused on improving teachers’ digital skills, such as digital literacy, online communication and collaboration, digital content creation and problem solving in virtual environments.

In conclusion, the implementation of digital technology architecture in university education, with tools such as VAS and cloud computing technology, is essential to improve the quality of education and teaching processes. This requires continuous training of teachers in the use of these tools and the reinvention of competences for the university teacher 4.0. Likewise, the continuity of research in the framework of academic intelligence supported by innovative technologies is essential to continue improving education in the digital era.

## Limitations

Despite the findings of this study, certain limitations have been identified that should be considered when interpreting the results. First, the research focuses exclusively on the architecture of digital technologies for teacher training, which may not fully reflect other factors that influence training effectiveness, such as individual motivation, predisposition towards digital learning or institutional support. Second, the reported increase in training satisfaction could be influenced by variables not controlled for during the study, such as teachers' previous level of technological competence or changes in the external educational environment.

Furthermore, the measurement of satisfaction was based on the subjective perception of the participants, which may be biased and not necessarily indicate an improvement in practical competence. The research was limited to a specific university setting, which may affect the generalisability of the results to other institutions with different academic cultures or technological resources. Finally, the study was conducted within a specific timeframe, and the long-term effects of training on the effective integration of digital technologies into teaching practice were not assessed. These limitations suggest the need for further studies to further explore the relationship between technology architecture and teacher training in virtual learning environments.

## Future directions and recommendations

Future directions for this study could include expanding the scope of the research to explore the integration of Education 4.0 in different educational contexts and cultures, which would allow for a broader understanding of how digital technologies can be adapted and applied globally. It would be beneficial to explore the long-term impact of digital literacy on teachers’ performance and adaptability in the changing educational landscape. In addition, future studies could focus on the development of specific digital literacy frameworks for teachers that address the emerging skills needed in the digital age.

Furthermore, in order to capitalise on the advances of Education 4.0, it is essential that institutions provide continuous training for teachers in new technologies and innovative pedagogies. Schools need to ensure a robust technology infrastructure to facilitate equitable access to digital resources. Collaboration across disciplines can enrich teaching practices, and continuous evaluation allows educators to refine their methods. Digital literacy should be a priority in teacher training, preparing teachers to guide students in a safe digital environment. In addition, adaptability in the face of rapid technological change is crucial, as is the active involvement of students in their digital learning. Finally, robust educational policies and a focus on digital safety and ethics will complete the preparation of teachers and students for the digital future.

## Data Availability

The data used in this research are available in a data repository. Zenodo: Survey on the implementation of digital technology architecture in teacher training. https://doi.org/10.5281/zenodo.10085049.
